# A Robust Approach for a Filter-Based Monocular Simultaneous Localization and Mapping (SLAM) System

**DOI:** 10.3390/s130708501

**Published:** 2013-07-03

**Authors:** Rodrigo Munguía, Bernardino Castillo-Toledo, Antoni Grau

**Affiliations:** 1 Department of Computer Science, CUCEI, University of Guadalajara, Av. Revolución 1500 Modulo “O” Col. Olimpica, Guadalajara 44830, Jalisco, Mexico; 2 Center for Research and Advanced Studies, CINVESTAV, Unidad Guadalajara, Av. del Bosque 1145, Col. El Bajío, Zapopan 45015, Jalisco, Mexico; E-Mail: toledo@gdl.cinvestav.mx; 3 Department of Automatic Control, Technical University of Catalonia, C. Pau Gargallo 5 Campus Diagonal Sud Edifici U., Barcelona 08028, Spain; E-Mail: antoni.grau@upc.edu

**Keywords:** monocular SLAM, mobile robotics, stochastic estimation, localization, mapping

## Abstract

Simultaneous localization and mapping (SLAM) is an important problem to solve in robotics theory in order to build truly autonomous mobile robots. This work presents a novel method for implementing a SLAM system based on a single camera sensor. The SLAM with a single camera, or monocular SLAM, is probably one of the most complex SLAM variants. In this case, a single camera, which is freely moving through its environment, represents the sole sensor input to the system. The sensors have a large impact on the algorithm used for SLAM. Cameras are used more frequently, because they provide a lot of information and are well adapted for embedded systems: they are light, cheap and power-saving. Nevertheless, and unlike range sensors, which provide range and angular information, a camera is a projective sensor providing only angular measurements of image features. Therefore, depth information (range) cannot be obtained in a single step. In this case, special techniques for feature system-initialization are needed in order to enable the use of angular sensors (as cameras) in SLAM systems. The main contribution of this work is to present a novel and robust scheme for incorporating and measuring visual features in filtering-based monocular SLAM systems. The proposed method is based in a two-step technique, which is intended to exploit all the information available in angular measurements. Unlike previous schemes, the values of parameters used by the initialization technique are derived directly from the sensor characteristics, thus simplifying the tuning of the system. The experimental results show that the proposed method surpasses the performance of previous schemes.

## 1. Introduction

Simultaneous localization and mapping (SLAM) is perhaps the most fundamental problem to solve in robotics in order to build truly autonomous mobile robots. SLAM deals with the way in which a mobile robot can operate in an *a priori* unknown environment using only on-board sensors to simultaneously build a map of its surroundings, which it uses to track its position. Robot sensors have a large impact on the algorithm used in SLAM. Early SLAM approaches focused on the use of range sensors, such as sonar rings and lasers; see [1,2]. Nevertheless, there are some disadvantages when using range sensors in SLAM: correspondence or data association becomes difficult, they are expensive, have a limited working range and some of them are limited to 2D maps. Additionally, they are computationally inefficient when the number of features is large (see [3,4] for a complete survey).

The aforementioned issues have motivated that recent works move towards the use of cameras as the primary sensor. Cameras have become more and more interesting for the robotic research community, because they provide a lot of information for data association, although this problem remains latent. Cameras are well adapted for embedded systems: they are light, cheap and power-saving. Using vision, a robot can localize itself using common objects, such as landmarks. Moreover, information about far landmarks can be obtained and used by the robot.

In this context, the six degrees of freedom (DOF) monocular camera case possibly represents the most difficult variant of SLAM; in monocular SLAM, a single camera can be freely moving in its environment representing the only input sensor to the system. On the other hand, while range sensors (e.g., laser) provide range and angular information, a camera is a projective sensor, which measures the bearing of image features. Therefore, depth information (range) cannot be obtained in a single frame. This fact has motivated the appearance of special techniques for feature initialization approaches to allow the use of bearing sensors, such as cameras, in SLAM systems. In this sense, the treatment of the features in the stochastic map, such as, initialization, measurement, *etc.*, is perhaps still the most important problem in monocular SLAM in order to improve robustness.

As computational power grows, an inexpensive monocular camera can be used to perform simultaneously range and appearance-based sensing, replacing typical sensors for range measurement, like laser and sonar rings, and for dead reckoning (encoders). Thus, a camera connected to a computer becomes a position sensor, which could be applied to different fields, such as robotics (motion estimation in mobile robots), wearable robotics (motion estimation for camera-equipped devices worn by humans), telepresence (head motion estimation using an outward-looking camera) or television (camera motion estimation for live augmented reality) [5].

The paper is organized as follows: Section 2 presents a summary of the related work and gives insights about the contributions of this work. Section 3 describes the proposed method in a detailed manner. In order to show the performance of the proposed scheme, an extensive set of simulation results are presented in Section 4. Final remarks are given in Section 5.

## 2. Monocular SLAM Techniques

Monocular SLAM has received a lot of attention recently, and it is closely related to the structure-from-motion (SFM) problem for reconstructing scene geometry. SFM techniques, which originally come from the computer vision research community, are sometimes formulated as off-line algorithms that require batch processing for all the images acquired in the sequence. Nevertheless, several recursive solutions to the SFM problem can be found in the literature. In this sense, one of the first works was presented in [6], assuming that the camera motion is known. On the other hand, in [7], a method to estimate motion from a known structure is proposed. In [8], both of the previous problems were addressed. Other SFM-based techniques are presented in [9,10]. Hybrid techniques, like the SFM-Kalman Filtering based on stereo-vision, have also appeared [11].

In [5,12], a real-time method is proposed based on the well-known Extended Kalman Filter (EKF) framework as the main estimation technique. In those works, a Bayesian partial initialization scheme for incorporating new landmarks is used. A separate particle filter, which is not correlated with the rest of the map, is used in order to estimate the feature depth prior to its inclusion in the map. Prior to those works, the feasibility of real-time monocular SLAM was first demonstrated in [13].

In [14], a FastSLAM-based scheme is proposed. In that work, the pose of the robot is represented by particles, and a set of Kalman filters refines the estimation of the map features. Particle filtering (PF) is a nonlinear estimation technique, which has been also utilized for computing the monocular SLAM solution. The advantage of PF is that it can deal with nonlinear non-Gaussian models. A method based on PF is presented in [15].

In SLAM, the potential bad effects of incorrect or incompatible matches are well known. In this case, some works have focused on improving the robustness of this kind of system by proposing techniques of data association. For instance, in [16], multi-resolution visual descriptors are used. In [17], the problem of relocalization after filter divergence is addressed. Also, different schemes to increase the number of features of the map to be managed have appeared [18,19]. In these works, the problem of closing the loop is also discussed. For more details about the problem of closing the loop, a comparison of techniques is presented in [20].

In [21,22], an approach, where the transition from partially to fully initialized features does not need to be explicitly tackled, is presented, making it suitable for direct use in the EKF framework in sparse mapping. In this approach, the features are initialized with respect to the first frame where they are observed, with an initial fixed inverse depth and uncertainty heuristically determined to cover the range from nearby to infinite; therefore, distant points can be coded.

The monocular SLAM techniques have also been applied to other interesting areas of related research. For instance, in [23], the application of monocular SLAM to support augmented reality insertions on medical endoscopic surgery is explored. Also, in [24,25], applications to navigation systems for autonomous aerial vehicles are considered.

So far, most of the available approaches for monocular SLAM are based on filtering methods. On the other hand, the availability of recent powerful hardware has motivated the development of the so-called Keyframe methods. These methods, as the proposed in [26], retain the optimization approach of global bundle adjustment, but computationally, must select only a small number of past frames to process. While Keyframe methods are shown to give accurate results when the availability of computational power is enough, filtering-based SLAM methods might be beneficial if limited processing power is available [27].

The work presented in this paper is motivated by the application of monocular SLAM to small autonomous vehicles. In this case, and due to limited resources commonly available in this kind of application, the filtering-based SLAM methods seem to be still more appropriate than Keyframe methods. Moreover, filtering-based methods are better suited for incorporating, in a simple manner, additional sensors to the system. In this sense, most robotics applications make use of multiple sensor inputs.

In the authors previous work [28], a monocular SLAM approach is proposed. In that work, the so-called delayed inverse-depth feature initialization method is used to initialize new features in the system. The method, which is based on the inverse depth parameterization, defines a single hypothesis for the initial depth of features by using a stochastic technique of triangulation. In this case, the new visual features detected, called candidate points, are considered to be added to the map until they achieve a minimum angle of parallax.

In delayed methods, a feature observed in the instant, *t*, is added to the map in a subsequent time, *t* + *k*. Usually, the delay is used in this kind of method for collecting information that allows initializing of the feature in a robust manner. On the other hand, the undelayed methods take advantage of the observation of the feature from the instant, *t*, to the system update. However, this updating step should be computed carefully; otherwise, this kind of method may present divergences, due to the possibility of ill-conditioned initial conditions, as in the case of the initial depth of landmarks.

The delayed method proposed in the authors previous work [28] has shown good results. Nevertheless, due to its delayed nature, this method does not take advantage of the full information provided by visual landmarks (from the instant *t* to *t* + *k*).

Also, in [28], in order to incorporate distant features into the map, a minimum base-line is determined by the position of the camera, when a feature was observed for the first time, and the current position of the camera. This base-line is used then as an additional threshold for considering a candidate point to be initialized as a new map feature. Distant features do not produce parallax, but are very useful for providing information about orientation. To obtain a proper performance of this method, the parameter of the minimum base-line must be heuristically tuned, depending on the particular application.

In the authors previous research [29], the idea of a concurrent initialization method was introduced for a simplified and simulated 3-DOF bearing-only SLAM context, taking benefit of complementary advantages for the undelayed and delayed methods. However, this method still presents some important drawbacks or limitations, such as: (i) the need of an intense tuning of the measurement error covariance matrix in order to properly incorporate angular information from partially initialized features, and (ii) the impossibility for extending the method in a straightforward manner to be used in a 6-DOF monocular SLAM context.

This paper considerably extends the authors previous works [28,29] by introducing a novel method for implementing a filtering-based monocular SLAM system that is robust and easy to tune. The main contribution consists in a novel scheme based in a two-step architecture, for the initialization and measurement of features. In this sense, the idea introduced in [29] based on the concurrent use of two kinds of feature maps for modeling the landmarks of the environment, is regained and extended for its application to a full 6-DOF monocular SLAM context. The most relevant contributions with respect to the previous works are:

- A novel measurement model based on epipolar constraints is presented. This model improves camera pose “collection” information from visual features when depth estimation is not yet well conditioned.
- The use of tables for storing information about previous states of the features (candidate points) is avoided. In this case, the stochastic technique of triangulation, used for estimating a hypothesis for the initial depth of features, is carried out over the current system state and covariance matrix.
- In order to improve the modularity and scalability of the method, the process for detecting and tracking visual features is fully decoupled from the main estimation process. In this case, a simple, but effective, scheme is proposed.
- A new camera motion model is presented. This model is used in order to simplify the implementation of the method, because their Jacobians are simpler with respect to previous models.

The architecture of the method presented in this work permits us to take advantage of the full information provided by the angular measurements of the camera, as the undelayed methods do. At the same time, the method permits us to deal with the features in a robust manner, as in the delayed methods. Moreover, the proposed method has been designed in order to avoid the use of some of the parameters that have to be heuristically tuned in previous approaches, such as: (i) initial depth and uncertainty of features in [21,22], (ii) minimum base-line in [28] or (iii) standard deviation of the bearing-sensor in [29]. Instead, most of the parameter values used in the proposed approach are derived directly from the sensor characteristics or, at least, are systematically derived. The reduction of the parameters to be tuned is an important feature of the contributed approach, because it improves the robustness of the system and simplifies its application under different conditions, avoiding the need of an extensive tuning.

## 3. Method Description

### 3.1. System Parameterization and Assumptions

The complete system state, *x*, consists of:

(1) $$x={\left(x_{v},y_{1},y_{2},\ldots ,y_{n}\right)}^{T}$$

where *x_v_* represents the state of a free camera moving in any direction in ℝ^3^ × *SO*(3). Features of the scene that are included into the system state, *x*, are defined as*y_i_*.

At the same time, *x_v_* is composed of:

(2) $$x_{v}={\left(q^{NC},\omega^{C},r^{N},v^{N}\right)}^{T}$$

where: *q^NC^* = (*q*_1_, *q*_2_, *q*_3_, *q*_4_)*^T^* represents the orientation of the camera with respect to the world (navigation) frame by a unit quaternion. The superscripts *N* and *C* denote magnitudes expressed in the navigation reference frame and in the camera reference frame, respectively A superscript *AB* denotes a reference frame, *B*, expressed with respect to the reference, *A. r^N^=* (*x_v_*, *y_v_*, *z_v_*)*^T^* represents the camera optical center position in Cartesian coordinates. *ω^C^* = (*ω_x_*, *ω_y_*, *ω_z_*)*^T^* and: *v^N^=* (*v_x_*, *v_y_*, *v_z_*)*^T^* denote linear and angular velocities, respectively.

**Figure 1.**
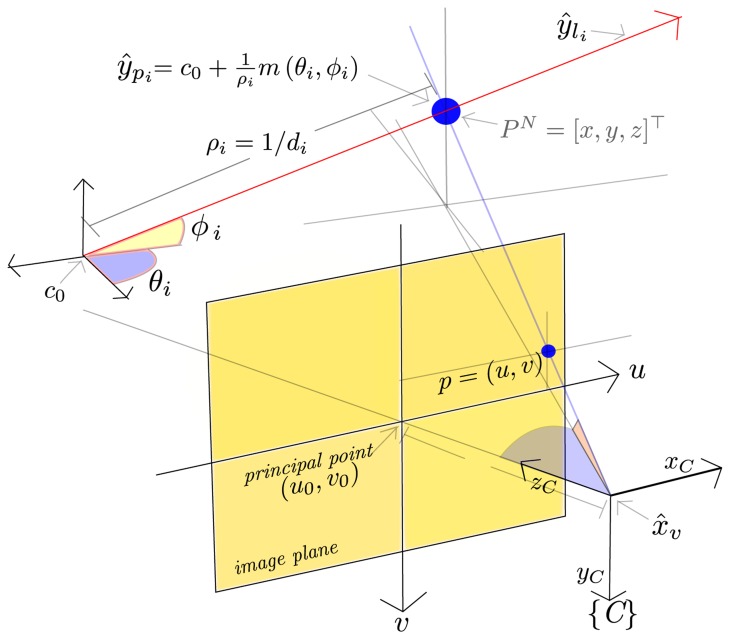
Feature parametrization.

Two kinds of features, *y_i_*, are considered (see Figure 1): *y_l_i__* and *y_pi_*, where:

(3) $$y_{l_{i}}={\left(x_{i},y_{i},z_{i},\theta_{i},\phi_{i}\right)}^{T}$$

models a 3D semi-line, defined on one side by the vertex, *c*_0_ = (*x_i_*, *y_i_*, *z_i_*)*^T^*, corresponding to the optical center coordinates of the camera when the feature was observed for the very first time and pointing to infinity on the other side, with azimuth and elevation, *θ_i_* and *ϕ_i_*, respectively. All the elements in Equation (3) are expressed with respect to the navigation reference frame, *N*.

On the other hand:

(4) $$y_{p_{i}}={\left(x_{i},y_{i},z_{i},\theta_{i},\phi_{i},\rho_{i}\right)}^{T}$$

models a 3D point, *P^N^*, located at:

(5) $$P^{N}=c_{0}+\frac{1}{\rho_{i}}m(\theta_{i},\phi_{i})$$

where *m*(*θ_i_*, *ϕ_i_*) is a directional unitary vector defined by:

(6) $$m(\theta_{i},\phi_{i})={\left(\cos\theta \sin\phi ,\sin\theta \sin\phi ,\cos\phi \right)}^{T}$$

and the point depth, *d_i_*, is coded by its inverse value, *ρ_i_* = 1/*d_i_*.

**Figure 2.**
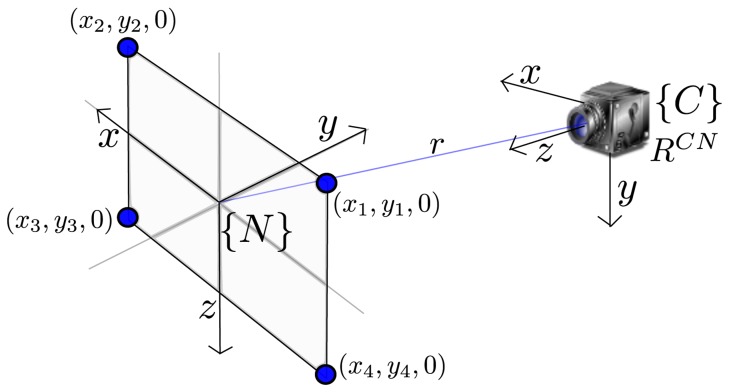
System initialization.

Figure 2 shows the relationship between the camera frame, *C*, and the navigation reference frame, *N*. In this work, the axes of the navigation frame, *N*, follow the North, East, Down (NED) convention.

In order to recover the metric scale of the world, at the beginning of the video sequence, it is assumed that four coplanar points are known. For instance, the dimensions of a black paper sheet over a white background. Besides the video sequence, this is the only extra information about the world provided to the system. It is also assumed that the intrinsic parameters of the camera are already known: (i) focal length *f*, (ii) principal point, *u*_0_, *v*_0_, and (iii) radial lens distortion, *k*_1_, …, *k_n_*. In this work, intrinsic parameters are estimated using [30]. More details about the system initialization are given in Section 3.2.

A central-projection camera model is assumed. In this case, the image plane is located in front of the camera's origin and on which a non-inverted image is formed (Figure 1.) The camera frame, *C*, is right-handed with the *z*-axis pointing to the field of view.

The ℝ^3^ ⇒ ℝ^2^ projection of a 3D point located at *P^N^* = (*x*, *y*, *z*)*^T^* to the image plane, *p* = (*u*, *v*), is defined by:

(7) $$u=\frac{x^{\prime }}{z^{\prime }}\;v=\frac{y^{\prime }}{z^{\prime }}$$

where *u* and *v* are the coordinates of the image point, *p*, expressed in pixels units, and:

(8) $$\left[\begin{array}{c} x^{\prime }\\ y^{\prime }\\ z^{\prime } \end{array}\right]=\left[\begin{array}{ccc} f & 0 & u_{0}\\ 0 & f & v_{0}\\ 0 & 0 & 1 \end{array}\right]P^{C}$$

being *P^C^* the same 3D point, *P^N^*, but expressed in the camera frame, *C*, by *P^C^* = *R^NC^* (*P^N^* − *r^N^*). *R^NC^* is the rotation matrix transforming from the camera frame to the navigation frame and is computed from the current camera quaternion, *q^NC^*.

Inversely, a directional vector, $h^{C}={\left(h_{x}^{C},h_{y}^{C},h_{z}^{C}\right)}^{T}$, can be computed from the image point coordinates, *u* and *v*:

(9) $$h^{C}(u,v)={\left[\frac{u_{0}-u}{f},\frac{v_{0}-v}{f},1\right]}^{⊤}.$$

The vector, *h^C^*, which is expressed in the camera frame, *C*, points from the camera optical center position to the 3D point location. Note that for the ℝ^2^ ⇒ ℝ^3^ mapping case, defined in Equation (9), depth information is lost.

### 3.2. System Initialization

In a robotics context, obtaining the metric scale of the world can be very useful. However, in monocular SLAM, the scale of the observed world cannot be obtained using only vision, and therefore, another sensor or the observation of a known dimension reference have to be used in order to retrieve the scale of the world.

In this work, the system metric initialization process is analogous to a classical problem in computer vision: the perspective of *n*-point (PnP) problem [31]. The PnP problem is to find the position and orientation of a camera with respect to a scene object from *n* correspondent points. In [31], it is demonstrated that, when the control points are coplanar, the perspective on PnP problem has a unique solution.

The problem consists in estimating two extrinsic camera parameters: the camera to navigation rotation matrix for camera orientation, *R^CN^*, and the translation vector for the position of the camera center, *r*, given four coplanar points with spatial coordinates, (*x_i_*, *y_i_*, *0*), *i* ∈ (1, ‥, 4), and their corresponding four undistorted image coordinates, (*i*, *j*), as shown in Figure 2. The methodology for estimating *R^CN^* and *r* is detailed in [28].

The initial system state, *x̂_ini_*, is formed by the initial camera state, *x̂_v_*_(_*_ini_*_)_, and the four initial features used for estimating the extrinsic camera parameters, namely:

(10) $${\hat{x}}_{\text{ini}}={\left(q_{\text{ini}}^{NC},\omega_{\text{ini}}^{C},r_{\text{ini}}^{N},v_{\text{ini}}^{N},{\hat{y}}_{1},{\hat{y}}_{2},{\hat{y}}_{3},{\hat{y}}_{4}\right)}^{T}$$

where $q_{\text{ini}}^{NC}$ is computed from *R^CN^*, using the corresponding rotation-matrix to quaternion transformation, $r_{\text{ini}}^{N}=r$, $\omega_{\text{ini}}^{C}=(0_{3\times 3})$, $v_{\text{ini}}^{N}=(0_{3\times 3})$. Each initial feature, *ŷ_i_*, for *i* = (1, ‥, 4) corresponds to each reference point, (*x_i_*, *y_i_*, 0), but parameterized, as in Equation (4).

### 3.3. Camera Motion Model

At every step, *k*, the camera system state, *x̂_v_*, is taken a step forward by the following unconstrained constant-acceleration discrete model:

(11) $$\{\begin{array}{l} q_{k+1}^{NC}=\left(\cos\left\|\text{w}\right\|{\text{I}}_{4\times 4}+\frac{\sin\left\|\text{w}\right\|}{\left\|\text{w}\right\|}\text{W}\right)q_{k}^{NC}\\ \omega_{k+1}^{C}=\omega_{k}^{C}+\Omega^{C}\\ r_{k+1}^{N}=r_{k}^{N}+v_{k}^{N}\Delta t\\ v_{k+1}^{N}=v_{k}^{N}+V^{N} \end{array}$$

In the model given by Equation (11), a closed form solution of *q̇* = 1/2(W)*q* is used to integrate the current velocity rotation, *ω^C^*, over the quaternion, *q^NC^*. In this case, ${\left({\text{w}}_{1},{\text{w}}_{2},{\text{w}}_{3}\right)}^{T}={\left(\omega_{k+1}^{C}\Delta t/2\right)}^{⊤}$, and:

(12) $$\text{W}=\left[\begin{array}{cccc} 0 & -{\text{w}}_{1} & -{\text{w}}_{2} & -{\text{w}}_{3}\\ {\text{w}}_{1} & 0 & -{\text{w}}_{3} & {\text{w}}_{2}\\ {\text{w}}_{2} & {\text{w}}_{3} & 0 & -{\text{w}}_{1}\\ {\text{w}}_{3} & -{\text{w}}_{2} & {\text{w}}_{1} & 0 \end{array}\right]$$

At each step, it is assumed that there is an unknown linear and angular velocity with zero-mean acceleration and an assumed covariance Gaussian process, *σ_a_* and *σ_ω_*, producing an impulse of linear and angular velocity, $V^{N}=\sigma_{a}^{2}\Delta t$ and $\Omega^{C}=\sigma_{\omega }^{2}\Delta t$.

It is also assumed that map features remain static (rigid scene assumption) so *x̂*_*k*+1_ = (*x̂_v_*_(_*_k_*_+1)_, *ŷ*_1(_*_k_*_)_, *ŷ*_2(_*_k_*_)_, …, *ŷ_n_*_(_*_k_*_)_)*^T^*. An extended Kalman filter (EKF) propagates the camera pose and the velocity estimates, as well as the feature estimates.

### 3.4. Feature Detection and Matching

Filtering-based methods commonly rely in active search techniques [32]. On the other hand, in this work, the feature tracking is decoupled from the main estimation process. In this case, a standard small base-line tracker is proposed for detecting and tracking visual features. For this purpose, the Kanade-Lucas-Tomasi Tracker (KLT) [33] is used, but any small base-line tracker can be used.

At the beginning of the video sequence, manual assistance is needed for selecting the four points corresponding to the initial metric reference. After this, detection of new features and their tracking are completely conducted by the KLT. At each frame, *k*, a list, *m*_(_*_k_*_)_ = (*u*_*d*_1__, *v*_*d*_1__, *u*_*d*_2__, *v*_*d*_2__, …*u*_*d*_*n*__, *v*_*d*_*n*__), of *n* measurements, (*u*_*d*_*i*__, *v*_*d*_*i*__), are obtained from the KLT and passed to the filter. Here, *u*_*d*_*i*__ and *v*_*d*_*i*__ indicate the distorted pixel coordinates for each feature, *i*.

Most of the time, the KLT produces good quality results; however, sometimes, false positives are also obtained (e.g., detection of landmarks running along edges). The above drawbacks can be mitigated by the use of batch validation techniques [34,35]. Nevertheless, this kind of technique commonly adds extra computational time. In this work, a simple and fast validation technique is proposed, which has been shown to give good results in combination with the KLT. This technique may be resumed as follows:

- When a visual feature, *i*, is considered to be added to the stochastic map in frame, *k*, then a *p* × *p* pixel window, *pw*_(k)_, around [*u*_*d*_*i*__, *v*_*d*_*i*__] is stored and related to the *i* feature.
- At every subsequent frame, (*k* + 1, *k* + 2, ‥*k* + *n*), a new *p* × *p* pixel window, *pw*_(_*_k_*_+_*_n_*_)_, around the current (*u*_*d*_*i*__, *v*_*d*_*i*__) position found by the KLT is extracted.
- The patch cross-correlation technique is applied between the current pixel window, *pw*_(_*_k_*_+_*_n_*_)_, and the stored pixel window, *pw*_(_*_k_*_)_. If the score obtained is higher than a threshold, then the current (*u*_*d*_*i*__, *v*_*d*_*i*__) position is assumed to be a valid measurement.

It is important to highlight that the modularity of the whole system has been considerably increased by means of decoupling the tracking process of visual features from the main estimation process. In this case, other alternatives for data association (e.g., [36]) could be plugged into to the system in a straightforward manner, replacing the technique described above.

### 3.5. Processing of Features y_li_

As stated before, depth information cannot be obtained in a single measurement when bearing sensors are used. To infer the depth of a feature, the sensor must observe this feature repeatedly as it moves freely through its environment, estimating the angle from the feature to the sensor center. The difference between those angle measurements is the parallax feature. Actually, parallax is the key that allows estimating feature depth. In the case of indoor sequences, a displacement of centimeters is enough to produce parallax; on the other hand, the more distant the feature, the more the sensor has to travel to produce parallax.

Delayed methods wait until the sensor movement produces some degrees of parallax, in order to initialize a feature in a robust manner. Nevertheless, features with unknown depth or with huge uncertainty in depth can still provide useful information, such as very far landmarks that will never produce parallax, but can improve camera pose estimates.

Features, *y_l_i__*, are intended to improve camera pose by “collecting” information from visual features when depth estimation is not yet well conditioned. The initialization and measurement of features, *y_l_i__*, is summarized as follows.

#### 3.5.1. Initialization of Features *y_l_i__*

When a visual feature is detected for the first time, at an instant, *k*, it is initialized in the system state as follows:

The undistorted pixel coordinates, (*u*_*u*_*i*__, *v*_*u*_*i*__), are obtained from (*u*_*d*_*i*__, *v*_*d*_*i*__), applying the inverse of an distortion model. In this work, the distortion model described in [30] is used.

A directional vector, *h^C^*, pointing from the camera optical center position (where the feature was observed for the first time) to the feature location is computed from (*u*_*u*_*i*__, *v*_*u*_*i*__) using Equation (9).

The directional vector, $h^{N}={\left(h_{x}^{N},h_{y}^{N},h_{z}^{N}\right)}^{T}$, expressed in the navigation frame, *N*, is obtained as: *h^N^* = *R^CN^h^C^*, where the rotation matrix transforming from camera to navigation frame is computed from the current camera quaternion, *q^NC^*.

The system state, *x̂*, is augmented with a new feature, *ŷ_l_i__*_(_*_new_*_)_ = *g_l_*(*x̂*_(_*_k_*_)_, *m*_(_*_k_*_)_), as:

(13) $$\left[\begin{array}{c} x_{i}\\ y_{i}\\ z_{i}\\ \theta_{i}\\ \phi_{i} \end{array}\right]=\left[\begin{array}{c} x_{v}(k)\\ y_{v}(k)\\ z_{v}(k)\\ \text{atan}2(h_{y}^{N},h_{x}^{N})\\ \text{acos}\left(\frac{h_{z}^{N}}{\sqrt{{\left(h_{x}^{N}\right)}^{2}+{\left(h_{y}^{N}\right)}^{2}+{\left(h_{z}^{N}\right)}^{2}}}\right) \end{array}\right]$$

where *x_v_*(*k*), *y_v_*(*k*), *z_v_*(*k*) represent the current camera optical center. The state covariance matrix, *P̂*_(_*_k_*_)_, is updated as follows:

(14) $${\hat{P}}_{\text{new}}=\nabla Y_{l}\;\left[\begin{array}{cc} {\hat{P}}_{\left(k\right)} & 0\\ 0 & {\text{I}}_{2\times 2\left(\sigma_{uv}^{2}\right)} \end{array}\right]\nabla Y_{l}^{⊤}$$

where ∇*Y_l_* is the Jacobian formed by the partial derivatives of the initialization equations, *g_l_*(*x̂*_(_*_k_*_)_, *m*_(_*_k_*_)_), with respect to both the system state, *x̂*, and the component, ${\text{I}}_{2\times 2}(\sigma_{uv}^{2})$. *σ_uv_* is the standard deviation for the measurement error defined in pixel units.

#### 3.5.2. Measurement of Features, *y_l_i__*

**Figure 3.**
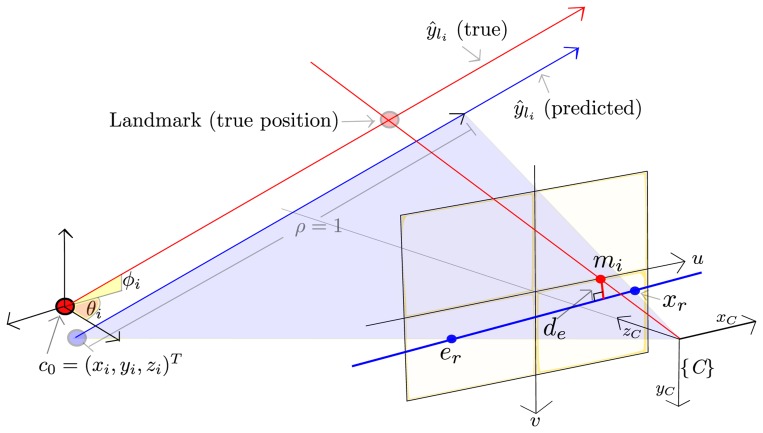
Model measurement for features, *ŷ_l_i__*.

If the camera moves from the location at which a feature, *y_l_i__*, is initialized, then this feature, which is modeled as a 3D semi-line, can be projected to the current image plane. The above projection, *l_r_*, is the 2D line, expressed in the image plane, defined by the epipole, *e_r_*, and the point, *x_r_*, (see Figure 3).

The epipole, *e_r_*, is computed by projecting the origin, *c*_0_ = (*x_i_*, *y_i_*, *z_i_*)*^T^*, of the feature, *ŷ_l_i__*, to the current image plane by Equations (7) and (8).

Using Equations (5), (7) and (8), the point, *x_r_*, is computed by projecting the 3D point defined by the feature, *ŷ_l_i__*, but assuming a depth equal to one (*ρ* = 1).

The epipolar constraint implies that new undistorted measurements of the landmark, *m_i_* = (*u_u_i__*, *v_u_i__*), should lie over the line, *l_r_*. Therefore, *d_e_*, which is the distance from the current measurement, *m_i_*, to the line, *l_r_*, is used as innovation error (*measurement–prediction*), in order to update the filter.

In this way, *d_e_* is computed as:

(15) $$d_{e}=\frac{\left\|\left(e_{r}-x_{r})\times (x_{r}-m_{i}\right)\right\|}{\left\|\left(e_{r}-x_{r}\right)\right\|}.$$

### 3.6. Processing of Features, y_p_i__

As stated before, depth information of landmarks can be inferred when the camera movement produces parallax. In this work, a stochastic technique of triangulation is used for computing the hypotheses of depth of features, *y_l_i__*. The idea is that features, *y_l_i__*, producing enough parallax, should be updated into the system state, *x̂*, as new features, *y_pi_*. The initialization and measurement of features, *ŷ_p_i__*, is summarized as follows:

#### 3.6.1. Initialization of Features *y_p_i__*

**Figure 4.**
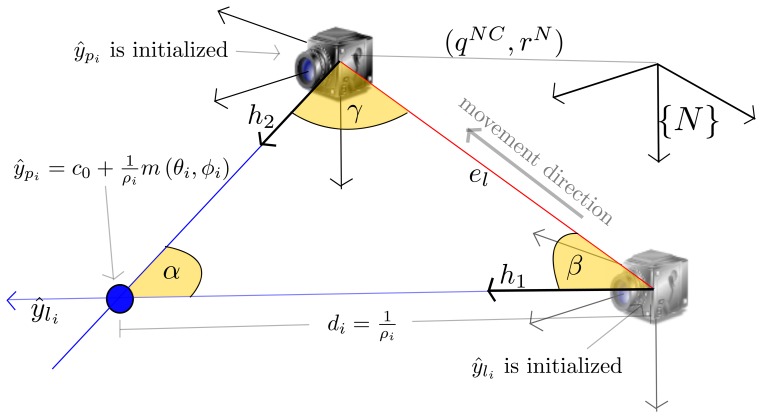
Representation for initialization process of features, *ŷ_p_i__*.

Every time a new measurement, *m_i_* = (*u_d_i__*, *v_d_i__*), is available for a feature, *ŷ_l_i__*, an estimation of depth, *d_i_* = *f*(*α_i_*, *γ*, *e_l_*), is computed (as can be seen in Figure 4):

(16) $$d_{i}=\frac{e_{l}\sin\gamma }{\sin\alpha }$$

where the parallax *α_i_* is computed by:

(17) $$\alpha_{i}=\pi -(\beta +\gamma )$$

and:

(18) $$\beta =\cos^{-1}\left(\frac{h_{1}\cdot e_{l1}}{\left\|h_{1}\right\|\left\|e_{l1}\right\|}\right)\;\gamma =\cos^{-1}\left(\frac{h_{2}\cdot e_{l2}}{\left\|h_{2}\right\|\left\|e_{l2}\right\|}\right)$$

and being *h*_1_ the normalized directional vector computed from Equation (6) using *θ_i_*, *ϕ_i_* is taken from *ŷ_l_i__*. *h*_2_ = *R^CN^h^C^* is the directional vector, pointing from the current camera optical center to the feature location. *h_C_* is computed from the undistorted pixel coordinates, (*u_u_i__*, *v_u_i__*) = *f*(*m_i_*), using Equation (9). *e_l_*_1_ is the epipolar line pointing from the origin, *c*_0_ = (*x_i_*, *y_i_*, *z_i_*)*^T^*, of the feature, *ŷ_l_i__*, to the current camera optical center, *r^N^*. *e_l_*_2_, and is the same epipolar line as *e_l_*_1_, but pointing in the opposite direction. *e_l_* = ║*e_l_*_1_║ = ║*e_l_*_2_║ is the magnitude of the epipolar line.

An estimate, $\sigma_{d_{i}}^{2}$, of the uncertainty of *d_i_* should also be computed using Equation (14), but now being ∇*Y_l_* the Jacobian formed by the partial derivatives of the triangulation equations, *d_i_= f*(*α_i_*, *γ*, *e_l_*), with respect to the system state, *x̂*. In the previous authors work [29], it is shown that only a few degrees of parallax are enough to reduce the uncertainty in depth estimations. Then, when the parallax is greater than a threshold (*α_i_* > *α_min_*), the feature, *ŷ_l_i__*, is updated as the feature of the type, *ŷ_p_i__*, by adding *ρ_i_* = 1/*d_i_* and $\sigma_{\rho_{i}}^{2}=(1/d_{i}^{4})\sigma_{d_{i}}^{2}$ to the system vector, *x̂*, and system covariance matrix, *P̂*:

(19) $${\left(x_{i},y_{i},z_{i},\theta_{i},\phi_{i}\right)}^{T}\Rightarrow {\left(x_{i},y_{i},z_{i},\theta_{i},\phi_{i},\rho_{i}\right)}^{T}.$$

If *j* corresponds to the index, in the system state, *x̂*, of the inserted *ρ_i_*, then $\sigma_{\rho_{i}}^{2}$is inserted into the (*j*, *j*) index of *P̂* in order to update the system covariance matrix, while all the rest of the elements of the inserted column, *j*, and row, *j*, are equal to zero.

Because the estimated depth, *d_i_*, varies considerably for low parallax, better results have been found by filtering *d_i_* over each *k* step (prior to its use in Equation (19)). If this is the case, an optional and simple extra linear Kalman filter (KF) can be used for this purpose. $\sigma_{d_{i}}^{2}$ can also be used as the measurement error of the extra filter. In this manner, *P̂* can be updated with the variance obtained from the extra KF, instead of $\sigma_{d_{i}}^{2}$.

#### 3.6.2. Measurement of Features *y_p_i__*

A feature, *y_p_i__*, is predicted to be measured at the *h_i_=* (*u_d_i__*, *v_d_i__*) pixel coordinates, using the following measurement prediction model, *h_i_= h*(*x̂_v_*, *ŷ_p_i__*).

First, the Euclidean representation, *P^N^*, of feature, *ŷ_p_i__*), is computed using Equation (5). Then, the undistorted pixel coordinates of the feature (*u_u_i__*, *v_u_i__*) are found using the central-projection camera model defined by Equations (7) and (8). Finally, the distorted pixel coordinates (*u_d_i__*, *v_d_i__*) are obtained from (*u_u_i__*, *v_u_i__*) applying the corresponding distortion model.

### 3.7. Map Management

A SLAM framework that works reliably locally can easily be applied to large-scale problems using methods, such as sub-mapping, graph-based global optimization [27] or global mapping [19]. Therefore, in this work, large-scale SLAM and loop-closing is not considered. Although, these problems have been intensively studied in the past.

Moreover, this work is motivated by the application of monocular SLAM in the context of visual odometry. When the number of features in the system state increases, then computational cost grows rapidly, and consequently, it becomes difficult to maintain the frame rate operation. To alleviate this drawback, old features can be removed from the state for maintaining a stable number of features and, therefore, to stabilize the computational cost per frame. Obviously, if old features are removed, then previous mapped areas cannot be recognized in the future. However, in the context of visual odometry, this fact is not considered as a problem. A modified monocular SLAM method that maintains the computational operation as stable can be viewed as a complex real-time virtual sensor, which provides appearance-based sensing and emulates typical sensors, such as laser for range measurement and encoders for dead reckoning (visual odometry) [19].

This present work deals with the approach of monocular SLAM as a virtual sensor. Therefore, if the number of features exceeds a specified threshold, then the algorithm removes older features that were left behind by camera movement in order to maintain a stable amount of features in the system and, thus, stable computation time.

## 4. Experimental Results

The proposed method was implemented using MATLAB® R2010b (MathWorks, Natick, MA, USA) in order to test its performance. Because the feature detection and tracking process is completely decoupled from the main estimation process (see Section 3.4), then either both (i) synthetic measurements or (ii) pre-processed measurements acquired by a camera can be used as the system input. In experiments, the following parameter values have been used: standard deviations for linear and angular acceleration: *σ_a_* = 1 *m*/*s*^2^, *σ_ω_* = 1 *rad*/*s*^2^, respectively; standard deviation for the measurement noise: *σ_uv_* = 1 *pixel*; and minimum parallax angle: *α_min_* = 5°.

### 4.1. Experiments with Synthetic Data (Simulations)

The proposed method is intended to exploit all the information available in angular measurements, but without sacrificing robustness. Features, *y_l_i__*, are representations of landmarks producing low or even null parallax (e.g., landmarks recently detected or located too far).

**Figure 5.**
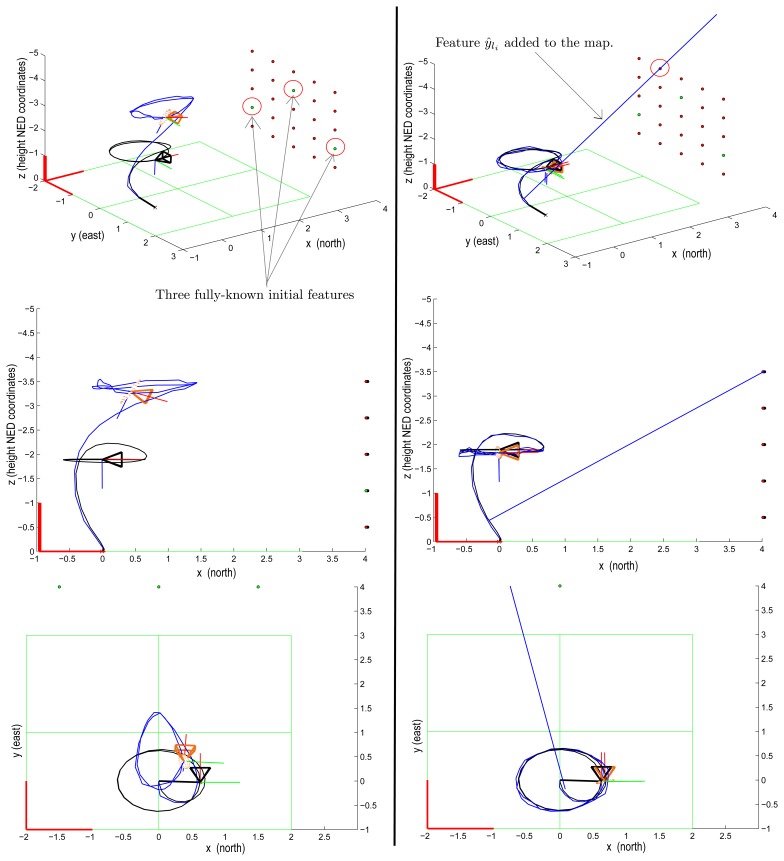
These simulations show the positive effect of the inclusion into the map of features with unknown depth, *y_l_i__*. In the first simulation (left plots), the map has been initialized with three landmarks whose location is perfectly known. In the second simulation (right plots), a single feature, *y_l_i__*, is added to the map. It can be noted that the estimated trajectory (in blue) is considerably better, when the feature, *y_l_i__*, is considered.

Figure 5 shows a simulation of a camera always seeing a “wall” of landmarks located at four meters from the origin. At the beginning of the simulation, the camera is moving from the floor to an elevation of two meters and then moving following a circular trajectory with a radius of one meter.

In the simulation, the map is initialized with three landmarks whose location is perfectly known (zero uncertainty). Nevertheless, in the plots to the left, it can be clearly appreciated that when only the three initial landmarks are considered, the estimated trajectory of the camera (blue line) differs substantially from the actual one (black line).

On the other hand, the plots on the right show the same simulation using the three initial landmarks, but in this case, a single random feature, *y_l_i__*, is also added to the map (blue line). It can be clearly appreciated that the inclusion of the feature, *y_l_i__*, is enough to produce the convergence of the estimates of the camera trajectory to the actual one. From the simulations, it can be concluded that features, *y_l_i__*, with unknown depth can also help to constraint the estimation of the camera location.

### 4.2. Experiments with Real Data

In order to test the proposed method with real data, several video sequences were acquired using a low cost camera. In this case, a Fire-i Unibrain monochrome webcam was used. This low-cost camera has an IEEE-1394 interface and an interchangeable wide angle lens. Video sequences with a resolution of 320 × 240 pixels acquired at 30 frames per second were used in the experiments. For each experiment, the point-based tracker was run over the video sequence, as is detailed in Section 3.4, and the outputs obtained from the tracker were stored in a plain text file. Finally, the MATLAB implementation was executed using the data previously stored as input.

**Figure 6.**
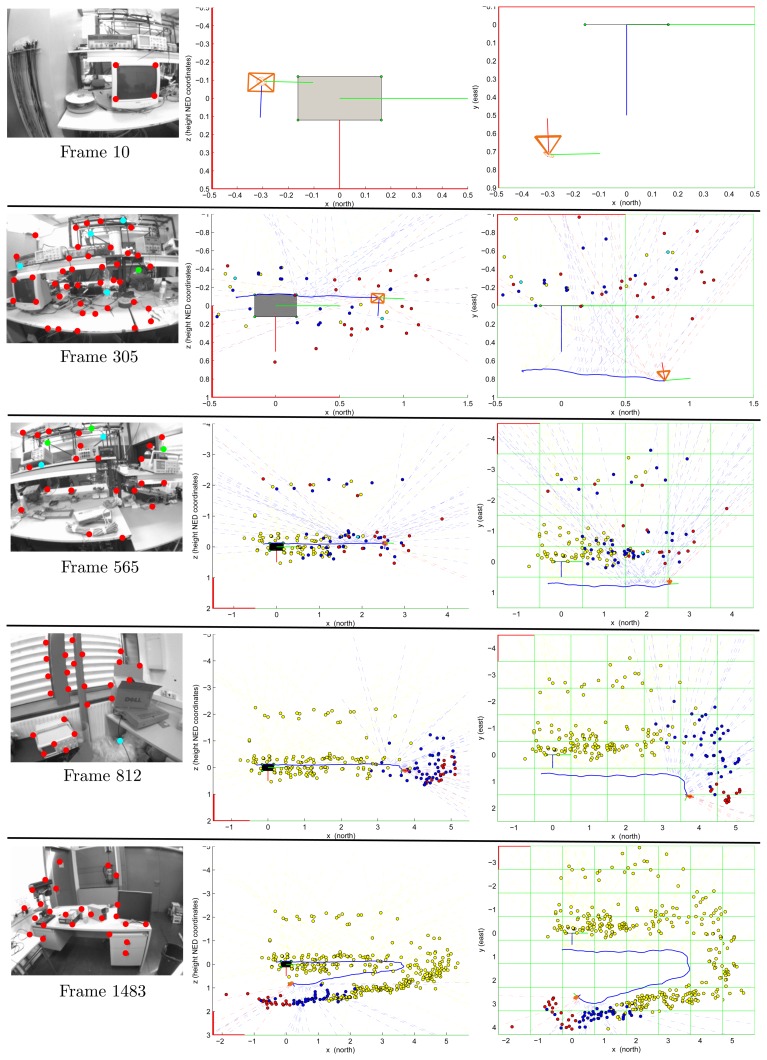
Input images (left column), camera trajectory and map estimations (z–x view; central column) (x–y view; right column) for a video sequence containing about 1,750 frames reordered in a laboratory environment. Frames 10, 305, 565, 812 and 1,483 are displayed.

Figure 6 shows the experimental results for a video sequence containing about 1,750 frames. This sequence has been recorded walking over a predefined cyclical trajectory inside a laboratory environment. In this experiment, the only reference for recovering the metric scale is a computer screen (Frame 10). The initial camera position and orientation is computed as described in Section 3.2. Note that the initial metric reference defines the origin of the navigation frame. In frame 305, the initial reference was left behind by the camera movement. In frame 812, it has totally completed its first turn to the defined trajectory. In frame 1,483, the camera is approaching its initial position. It is important to note that the experimental environment is challenging, due to the complexity of the structure, the huge variety of objects and different lighting conditions along the trajectory. In this case, occlusions and false visual landmarks are very common.

For the input images displayed in Figure 6: (i) points in red indicate feature points currently used as measurements, (ii) points in cyan indicate matches rejected by the simple validation procedure described in Section 3.2 and (iii) points in green indicate new visual features to be included in the map. For the plots illustrating the outputs of the proposed method, features, *y_l_i__* and *y_p_i__*, are indicated, respectively, by doted lines and points. The color code used is as follows: (i) features currently used as measurements are indicated in red, (ii) features contained in the system state, but rejected as measurements by the validation procedure are indicated in cyan, (iii) features still contained in the system state, but left behind by the movement of the camera (so, not used for updating the system state) are indicated in blue and (iv) old features deleted from the system state in order to maintain stable computational cost are indicated in yellow (see Section 3.7). Observe that several features, *y_l_i__*, were initialized, but never were updated as features, *y_p_i__*. The scale of the plots is in meters.

In order to compare the performance of the proposed method with respect to related works, an implementation of the popular Undelayed Inverse Depth (UID) method [21,22] was executed over the same databases that were also used for testing the proposed method. The first database corresponds to the previously described test; experiment (a). The second database, experiment (b), corresponds to a video sequence of 790 frames acquired in a desktop environment using a Microsoft LifeCam Studio webcam. This low cost camera has a USB interface and wide angle lens. It is capable of acquiring HD color video, but in the present experiments, gray level video sequences with a resolution of 424 × 240 pixels at 30 frames per second were used. In experiment (b), the camera was moved from left to right, following a trajectory similar to the curved front edge of a desktop.

In the experiments, the same values for parameters *σ_a_*,*σ_ω_*,*σ_uv_*, were used for both methods. In the case of the UID method, in order to achieve an initial convergence, the parameter of minimum-depth was heuristically tuned according to the environment conditions of each experiment. In order to improve the impartiality of the comparison, exactly the same four visual features (see Section 3.2) were used in both methods, for recovering the metric scale of the world. It is important to highlight that one of the benefits of decoupling the detection and tracking of visual features from the main estimation process lies in the fact that the comparatives between methods could be more impartial. For example, it is well known that the performance of an excellent SLAM algorithm can be severely affected for a few cases of spurious data association.

**Figure 7.**
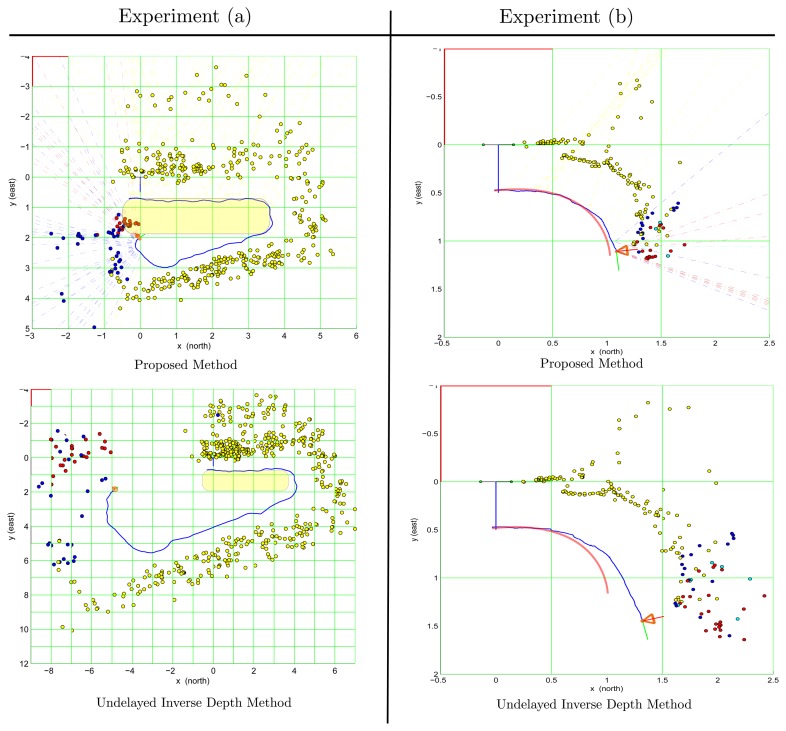
An x–y view corresponding to the camera trajectory and map estimates obtained from two different test cases. The right column shows the results from experiment (**a**), and the left column shows the results from experiment (**b**). In both cases, upper plots show the estimates obtained with the proposed method, whereas lower plots show the estimates obtained with the Undelayed Inverse Depth (UID) method. In experiment (a), the real path is described approximately by a rectangle with rounded edges. In experiment (b), the real path is indicated by a red curved line.

Figure 7 shows the final results obtained at the end of the trajectory from both tests: experiment (a) (left column) and experiment (b) (right column). In experiment (a), the real path followed when the video sequence was recorded is described approximately by a rectangle with rounded edges. In experiment (b), the real path is indicated by a red curved line.

In experiment (a), the results obtained with the UID method present a considerable degradation of the scale of the map and trajectory along time. On the other hand, with the proposed method, the consistency of the map and trajectory are much better preserved. In both cases, a clear degeneration in the estimated trajectory, and, therefore, in the map, happens when the camera is turning the curves (mainly in the second curve). This is the typical case when there are no far features to obtain enough information for the orientation. In this case, and without underestimating the effects of the drift induced in the curves, the 3D structure of the environment was recovered reasonable well with the proposed method, compared with the results obtained with the UID method.

In experiment (b), in the case of the UID method, it can be appreciated that the estimated trajectory of the camera and the map began to present a considerably degradation at the middle of the followed path. On the other hand, with the proposed method, the estimated trajectory was almost perfectly recovered for this test case.

Tables 1 and 2 show, respectively, the execution time for experiment (a) and experiment (b). As stated above, the experimental results were obtained from a non-optimized MATLAB implementation of both methods. The code was executed over a laptop with an Intel i5 M480 processor. In both experiments, features that were not matched in more than 30 consecutive frames were removed from the system state in order to maintain stable computation time (see Section 3.7). Tables 1 and 2 also show the mean and standard deviation for features maintained in the system state under the above condition. Based on the results displayed in Tables 1 and 2, especially regarding the number of features maintained in the system state, it can be inferred that real-time execution should be easily achieved by means of an optimized (i.e., C++) implementation. In this sense, from [5], real-time performance could be achieved for about 100 features in the system state in an EKF-based method. It is important to note that this proposed method exhibits a slightly higher computational cost compared with the UID method. Nevertheless, this extra cost is seen as being very acceptable, compared with the increase of performance earned with the proposed method.

## 5. Conclusions

In this work, an approach for implementing a filtering-based monocular SLAM system has been presented. The main contribution is to propose a novel and robust scheme for initializing and measuring the visual features. The proposed method is based on a two-stage technique, which is intended to exploit all the information available in angular measurements in a robust manner.

Two kind of features are incorporated to the stochastic map: features *y_l_i__* are intended to improve camera pose by “collecting” information from visual features for which depth estimation is still not well conditioned (such as landmarks recently detected or located too far). Features, *y_p_i__*, are representations of landmarks that are producing enough parallax in order to infer their depth.

Filtering-based methods commonly rely on active search techniques for detecting and matching visual features. Nevertheless, in this work and due to scalability purposes, the tracking process is completely decoupled from the main estimation process. In this case, a small base-line tracker is used together with a simple validation technique.

This work is motivated by the application of monocular SLAM in the context of visual odometry. In this context, old features are removed from the map in order to maintain a stable computational operation time. In such a case, this kind of monocular SLAM system can be viewed as a complex virtual sensor, which provides appearance-based sensing and emulates typical range sensors or encoders for dead reckoning. Therefore, the loop-closing problem is not considered in this work. Nevertheless, a SLAM framework that works reliably locally can be easily applied to large-scale problems.

Experiments with real data, as well as with simulated data have been carried out in order to validate the performance of the proposed method. The simulations clearly show the benefits of the inclusion into the map of features with unknown depth. Nevertheless, unlike some undelayed methods, any heuristic hypothesis is not made (or is not needed) about the initial depth of features. In this case, the value of parameters are derived directly from the sensor characteristics, thus simplifying the tuning of the system and improving the robustness of the system to different initial conditions.

For comparative purposes, the proposed method and the UID method were both executed over the same table of measurements obtained from different video sequences captured with low cost cameras. The results show that the consistency of the estimated map and trajectory are much better preserved with the proposed method. Based on the experimental results, it is suggested that the proposed method could be a robust alternative for implementing filter-based monocular SLAM systems.

## Figures and Tables

**Table 1.** Experiment (a): execution time and number of features into the system state.

Methods	Total Time (s)	Time per Frame (ms)	Number of Features
Proposed Method	89.5	53.1 ±11.5*σ*	40.8 ±7.5*σ*
UID Method	74.9	44.4 ±10.6*σ*	40.9 ±7.6*σ*

**Table 2.** Experiment (b): execution time and number of features into the system state.

Methods	Total Time (s)	Time per Frame (ms)	Number of Features
Proposed Method	26.8	34.5 ±19.4*σ*	30.4 ±15.2*σ*
UID Method	21.4	27.6 ±15.6*σ*	30.3 ±15.2*σ*
